# Passive Transfer of Animal-Derived Polyclonal Hyperimmune Antibodies Provides Protection of Mice from Lethal Lassa Virus Infection

**DOI:** 10.3390/v15071436

**Published:** 2023-06-26

**Authors:** Lisa Oestereich, Helena Müller-Kräuter, Elisa Pallasch, Thomas Strecker

**Affiliations:** 1 Department of Virology, Bernhard-Nocht Institute for Tropical Medicine, 20359 Hamburg, Germany; oestereich@bni-hamburg.de (L.O.); elisa.pallasch@bnitm.de (E.P.); 2 German Center for Infectious Research (DZIF), Partner Site Hamburg-Lübeck-Borstel-Riems, 20359 Hamburg, Germany; 3 Institute of Virology, Philipps University Marburg, 35043 Marburg, Germany; helena.mueller@staff.uni-marburg.de

**Keywords:** Lassa mammarenavirus, Lassa fever, arenavirus, virus-like particle, neutralizing antibodies, in vivo protection

## Abstract

Background: Lassa virus (LASV) can cause severe acute systemic infection in humans. No approved antiviral drugs or vaccines are currently available. Antibody-based therapeutics are considered a promising treatment strategy in the management of LASV disease. Methods: We used chimeric Ifnar^−/−^ C57BL/6 (Ifnar^−/− Bl6^) mice, a lethal LASV mouse model, to evaluate the protective efficacy of polyclonal antibodies purified from sera of rabbits hyperimmunized with virus-like particles displaying native-like LASV glycoprotein GP spikes. Results: Polyclonal anti-LASV GP antibodies provided 100% protection against lethal LASV infection in a pre- and post-exposure treatment setting and prevented LASV disease. Treatment also significantly lowered viremia level and virus load in organs. When treatment was initiated at the onset of symptoms, the hyperimmune antibodies provided partial protection and increased the survival rate by 80%. Conclusions: Our findings support the consideration of animal-derived hyperimmune antibodies targeting GP as an effective treatment option for highly pathogenic LASV.

## 1. Introduction

Lassa virus (LASV), a mammarenavirus belonging to the Arenaviridae family, is the human Lassa fever (LF) causative agent, which is a life-threatening systemic viral febrile illness endemic in West Africa. Continued large LF outbreaks have mainly been reported in Nigeria with a case fatality rate of up to 30% [1,2]. Acute kidney failure and neurological complications are frequently observed in critically ill LF patients [3]. Off-label use of ribavirin has shown partial efficacy [4]; however, licensed vaccines or specific therapeutics remain unavailable. Passive immunotherapy is a promising disease treatment strategy. While early clinical studies on convalescent plasma therapy in LF patients have shown varied results [5], monoclonal antibodies generated from LF survivors’ memory B cells provided complete protection against LF in a nonhuman primate model, even when administered at the advanced disease stages [6], demonstrating antibody-based therapeutic efficacy. The immunogenicity of virus-like particles (VLPs) expressing glycoprotein GP spikes from the prototypic LASV strain Josiah has been successfully demonstrated. Multiple immunizations of rabbits with the VLPs induced LASV GP-specific antibodies with broadly reactive neutralizing activity [7]. Here, the prophylactic and therapeutic in vivo capacity of these polyclonal antibodies against LASV GP were evaluated in a lethal mouse model of LASV infection.

## 2. Materials and Methods

### 2.1. Production of LASV GP VLPs Used for Immunization

The generation of LASV GP-induced virus-like particles (VLPs) has been described previously [8]. Briefly, Madin–Darby canine kidney strain II cells (MDCK II, ATCC CCL-34) stably expressing LASV GP (strain Josiah) were maintained in modified Eagle’s medium (MEM; Sigma-Aldrich, St. Louis, MO, USA) supplemented with 10% (*v*/*v*) fetal bovine serum (FBS; Thermo Fisher Scientific, Waltham, MA, USA), 2 mM L-glutamine (Sigma-Aldrich), and 50 U/mL penicillin and 50 µg/mL streptomycin (Sigma-Aldrich). For VLP production, cells were seeded in 175 cm^2^ tissue culture flasks and cultured at 37 °C for 4 days. The cell culture medium was then replaced by MEM supplemented with 2% FBS, 2 mM L-glutamine, and 50 U/mL penicillin and 50 µg/mL streptomycin, and the cells were incubated for an additional 4 days. Cell culture supernatants were harvested and clarified twice by centrifugation at 3345× *g* for 10 min at 4 °C. VLPs were then pelleted by ultracentrifugation through a 20% (*w*/*v*) sucrose cushion for 1.5 h at 110,000× *g* and 4 °C. The pellets containing VLPs were resuspended in sterile phosphate-buffered saline buffer solution and stored at 4 °C prior to use. The total protein concentration of the VLP preparations was determined using Pierce BCA Protein Assay Kit (Thermo Fisher Scientific). For anti-LASV GP antibody production, New Zealand White rabbits (Oryctolagus cuniculus; purchased from Charles River Laboratories, Wilmington, MA, USA) were immunized intramuscularly with 300 µg of LASV GP VLPs mixed in a 1:1 ratio (*v*/*v*) with Sigma Adjuvant System, and boosted 4, 7 and 10 weeks later. Final blood samples were collected on day 77 [7].

### 2.2. Purification and Functional Activity of Polyclonal Anti-LASV GP Antibodies

Total immunoglobulin G (IgG) was purified from rabbit antisera using the Antibody Serum Purification Kit (Protein A) from Abcam, according to the manufacturer’s instructions. The antibody concentration was determined using Pierce BCA Protein Assay Kit (Thermo Fisher Scientific). The purity of IgG preparations was assessed by 12% SDS-PAGE and SYPRO Ruby protein gel staining (Thermo Fisher Scientific) (Supplementary Figure S1A,B). The functional activity of purified IgG against LASV GP was controlled by enzyme-linked immunosorbent assay (ELISA) and antibody neutralization using replication-competent recombinant vesicular stomatitis virus (VSV) expressing LASV strain Josiah GP (VSVΔG/LASVGP) as previously described [7] (Supplementary Figure S1C,D).

### 2.3. Ethics Statement for LASV Challenge Study

This study was conducted in strict accordance with the recommendations of the German Society for Laboratory Animal Science under the supervision of a veterinarian. The protocol was approved by the Committee on the Ethics of Animal Experiments of the City of Hamburg (permit no. N061/21). All efforts were made to minimize the number of animals used for the experiments and suffering of the animals during the experiments. All staff performing animal experiments had passed an education and training program aligned with category B or C of the Federation of European Laboratory Animal Science Associations. The animal experiments in this study are reported in accordance with the ARRIVE guidelines.

### 2.4. Generation of Chimeric Mice

Ifnar^−/−^ (B6(Cg)-Ifnar1^tm1.2Ees^/J) and CD45.1^+^ congenic Ly5.1 (B6.SJL-Ptprc^a^ Pepc^b^/BoyJ) mice were obtained from Charles River and bred in the specific-pathogen-free animal facility at BNITM. The 8–12-week-old male and female Ifnar^−/−^ mice were irradiated and transplanted with bone marrow from Ly5.1 mice as described [9]. Mice were randomly assigned to cages (3–5 mice and 2–3 mice per cage for females and males, respectively) after the transplantation and kept in these groups for the experiments. At 6–8 weeks post-transplantation, engraftment was confirmed by staining peripheral blood lymphocytes for CD45.1 (from donor bone marrow) and CD45.2 (from the remaining Ifnar^−/−^ bone marrow). Only mice with an engraftment >80% were used for the experiments (obtained in 100% of tested mice). In total, n = 35 animals were used in this study and included in the analysis.

### 2.5. LASV Infection Experiments

Mice were kept in individually ventilated cages with a 12-h day/night cycle and ad libitum access to food and water. Cage enrichment was provided. Mice were transferred to the animal room in the BSL4 laboratory one week prior to infection to allow for acclimatization to the laboratory. Handling of the mice was performed by only one experienced scientist to reduce stress. Owing to the low number of cages (n = 8) and the short duration of the experiment (21 days), no further measures were employed to reduce confounders.

Mice were infected intraperitoneally with 1000 focus-forming units (FFU) of LASV strain Josiah (GenBank AY628203). Daily monitoring included weight and temperature measurement, as well as body scoring (based on behavior, appearance, and consciousness). An accumulative disease score was calculated daily to allow euthanasia after reaching humane endpoint criteria (details are provided in the Supplementary Table S1). Scoring frequency was increased to twice daily after the onset of symptoms; the n = 3 mice per group were euthanized on day 11 or 12 post-infection for organ harvest and n = 5 (treatment group) or 6 (isotype control group) mice were followed longitudinally until day 21 post-infection or until humane endpoint criteria were reached.

Survival, disease progression, and virus titers of the three different treatment groups (n = 8 mice per group) were compared with an isotype control antibody-treated group (n = 9 mice). Group size was chosen to allow the detection of 20% difference in survival rate. Complete cages were chosen per treatment group; no further randomization or blinding of the groups was performed. Mice were intraperitoneally (5 mL/kg) treated with purified 10 mg/kg polyclonal anti-LASV GP IgG (Supplementary Figure S1) or isotype control antibody at the following time points: pre-infection group −1 h and d1; post-infection group 1, d1, d3, and d5; post-infection group 2, d6, d8, and d10; isotype control group, d1, d3, and d5. Antibodies were diluted in sodium phosphate/sodium chloride buffer.

Blood from the tail vein was taken from all animals on days 3, 6, 9, 14, 17, and 21. Organs and heart blood were taken from all animals euthanized in accordance with the experimental study protocol on day 11 or 12, and all from animals that reached humane endpoint criteria or at the end of the experiment on day 21. Organs were homogenized in 1 mL DMEM without additives with Lysing Matrix D microspheres (MP Biomedicals, Irvine, CA, USA) in a bead mill. Viremia and virus titers in organs were determined using an immunofocus assay. Survival rates were plotted as a Kaplan-Meier survival curve and differences in survival rates were determined using the Mantel-Cox log-rank test. Differences in organ titers were calculated based on the non-parametric Mann-Whitney test. All graphs and statistical analysis were performed with the GraphPad Prism 9 software.

## 3. Results

**Figure 1:**
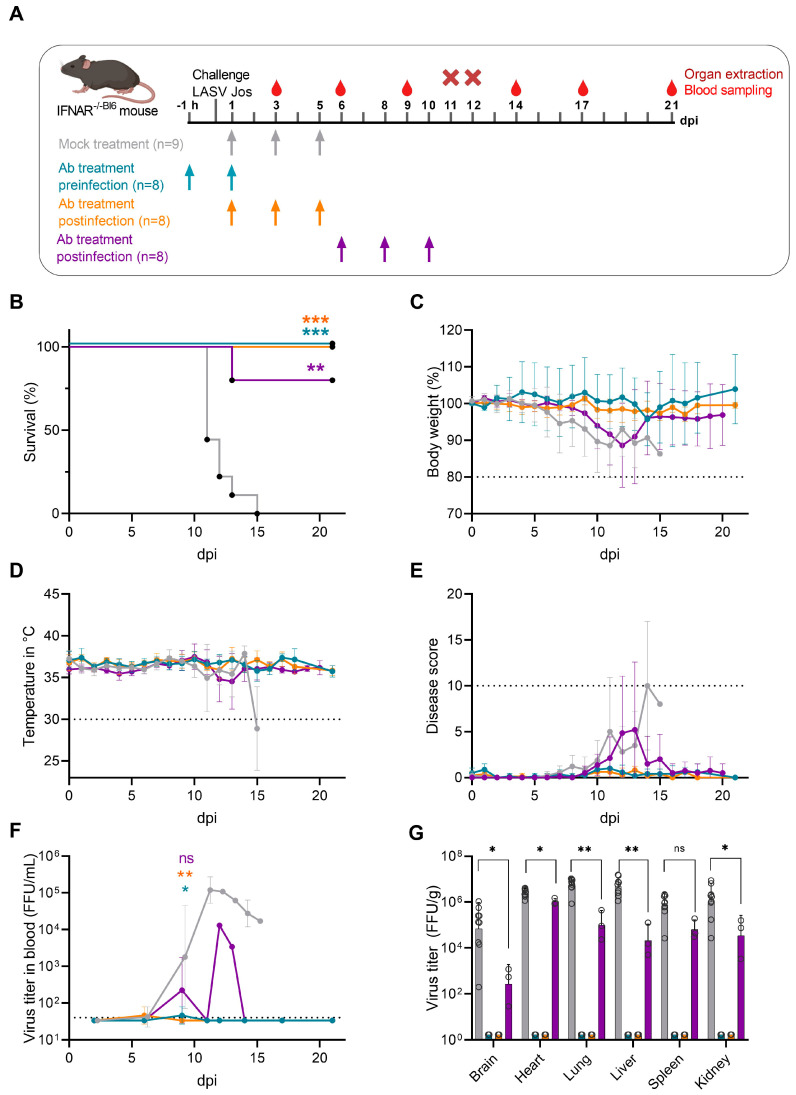
In vivo efficacy of polyclonal hyperimmune IgG specific for LASV glycoprotein. (**A**) Experimental treatment scheme to evaluate the effect of anti-LASV GP hyperimmune IgG as prophylactic and therapeutic treatment strategy in a lethal mouse model of LF. Chimeric Ifnar^−/− Bl6^ mice were infected intraperitoneally with 1000 FFU of LASV strain Josiah. Hyperimmune globulins (10 mg/kg) (n = 8 per group) or non-related isotype control (10 mg/kg) (n = 9) were administered intraperitoneally (5 mL/kg) at indicated time points before and after virus challenge. Blood and organ samples were collected at indicated time points post-infection for virological assessment. (**B**) Kaplan-Meier survival curve for the three treatment groups compared with mock-treated animals. Survival was evaluated by Mantel-Cox log-rank test using GraphPad Prism; ** *p* < 0.01, *** *p* < 0.001. (**C**) Relative weight change. (**D**) Core body temperature in °C. (**E**) Clinical scores of animals after challenge. (**F**,**G**) Blood viremia and viral titers of organs were assessed by immunofocus assay. Shown are the mean ± standard deviation. Significance was evaluated by Mann-Whitney test using GraphPad Prism; * *p* < 0.05, ** *p* < 0.01, ns: not significant. Euthanasia criteria for bodyweight, temperature and clinical score, and the limit of detection for viremia, are depicted as a dashed line. Dpi, days post-infection; FFU, focus-forming units; LASV, Lassa virus; IgG, immunoglobulin G.

We performed passive antibody transfer experiments in Ifnar^−/−^ mice transplanted with bone marrow from wild-type C57BL/6 mice (Ifnar^−/−Bl6^), a well-established animal model that recapitulates key characteristics of severe LF [9,10]. Here, three experimental treatment settings were designed, including pre-exposure (two-dose regimen) and post-exposure prophylaxis (three-dose regimen) as well as therapeutic intervention during the advanced infection stage (three-dose regimen) with n = 8–9 mice per group (Figure 1A). LASV-infected control animals rapidly lost weight and displayed disease signs from 6 dpi, and between 11 and 15 dpi all animals reached humane endpoint criteria and were euthanized (mean time to death: 11 dpi) (Figure 1B–E). Control animals developed high viremia and virus titers in organs.

At 9 dpi, mock-treated animals exhibited a mean plasma virus titer of 1.9 × 10^4^ FFU/mL and peaked on 11 dpi at 1.5 × 10^5^ FFU/mL (Figure 1F). High viral titers in the control animals’ brain, heart, lung, liver, spleen, and kidney between 2.76 × 10^5^ FFU/mL and 6.6 × 10^6^ FFU/mL were found (Figure 1G), indicating systemic spread. Pre-exposure prophylaxis with 10 mg/kg immunoglobulins at 1 h prior to virus challenge followed by a second antibody dose 24 h later protected all animals from severe LASV infection. When we evaluated the ability of anti-LASV GP hyperimmune IgG to provide post-exposure protection against lethal LASV infection, all mice survived when the antibodies were administered at 1, 3, and 5 dpi (Figure 1B). Both pre-exposure and post-exposure prophylaxis groups did not develop any clinical illness signs and did not exhibit temperature changes or lose weight (Figure 1C–E). Viremia was detected at very low levels 6 dpi, and on day 11–12 pi, no infectious virus could be recovered in the organs from the treated animals (Figure 1F,G). This suggested that antibody-based intervention in the early stage of infection efficiently neutralized and cleared infectious LASV. We then assessed whether the hyperimmune IgG antibodies also exerted therapeutic potential when administered to mice at an advanced infection stage. Therefore, treatment was initiated at 6 dpi, when mock-treated animals started to lose weight. The animals received two additional therapeutic antibody doses at 8 and 10 dpi, respectively. Here, 4/5 animals, that were followed longitudinally, survived and reached the study endpoint at 21 dpi without showing weight loss or clinical illness signs. One animal reached human endpoint on day 13 pi and was sacrificed. The peak virus titer in this animal’s blood was 3.4 × 10^4^ FFU/mL. In the animals that survived following antibody treatment, low or no viremia was detected at any time point after treatment. While virus was detectable in all organs taken at 11–12 dpi, titers in the treatment group compared to the control group were significantly lower in all organs except the spleen. No viremia rebound was observed in any surviving animals at 14, 17 and 21 dpi.

## 4. Discussion

Passive immunization with animal-derived hyperimmune antibodies has been shown to be effective in the prevention and therapy of various infectious diseases, including highly pathogenic viruses, such as Ebola virus, Zika virus and SARS-CoV-2 [11,12,13,14]. Here, the protective in vivo efficacy of polyclonal antibodies from rabbits hyperimmunized with LASV GP VLPs in a lethal mouse LASV infection model was demonstrated. Mice were completely protected by the hyperimmune IgG when administered before or soon after the LASV challenge and partially protected when treated at the onset of symptoms. Prophylactically treated animals did not develop any clinical disease signs or show body weight and body temperature changes. All treated animals had very low or undetectable viremia at all-time points analyzed. In animals having treatment that started late, outcome and disease severity correlated with effective viremia control, which is in line with observations in humans [15]. Our results indicated that the hyperimmune IgG can provide protection against lethal LASV challenge by effectively controlling virus replication. Fatal LF cases are often characterized by multiorgan failure in the terminal infection stage. It is encouraging that prophylactic treatment with polyclonal neutralizing hyperimmune IgG prevented LASV spread to secondary organs, as no infectious virus could be recovered from any tested organs obtained from treated animals. Future studies are needed to address if the hyperimmune IgG against LASV GP can induce sterile protection. Notably, even when treatment was commenced at 6 dpi, the hyperimmune IgG provided partial protection and increased survival rate by 80%. Optimizing antibody doses and treatment regimens at advanced infection stages may further increase the number of surviving animals. LASV exhibits substantial genomic diversity and is currently grouped into seven distinct phylogenetic lineages. Our previous in vitro studies showed broadly reactive neutralizing activity of the polyclonal anti-GP antibodies against representatives from five different LASV lineages [7]. It will be interesting to determine whether the hyperimmune IgG can protect against infection of antigenically divergent LASV strains.

Animal-derived antibodies may pose a safety concern due to their immunogenicity. However, advances in purification techniques and manufacturing processes significantly reduce the incidence of adverse reactions to animal immunoglobulins. Various animal-derived antibody products have received regulatory approval as therapies, such as, for example, snakebite envenoming, digoxin poisoning, and botulism [16]. Anti-thymocyte globulin preparations of rabbit origin are currently licensed for the prophylaxis and treatment of renal allograft rejection [17].

In conclusion, we provide encouraging in vivo results of heterologous animal-derived polyclonal hyperimmune antibodies as a cost-effective alternative to human-derived monoclonal antibodies for use in passive immunotherapy against highly pathogenic LASV.

## Data Availability

The data presented in this study are available on request from the corresponding author.

## Supplementary Materials

The following supporting information can be downloaded at: https://www.mdpi.com/article/10.3390/v15071436/s1, Figure S1: Purification and functional analysis of anti-LASV GP antibodies. Table S1: Clinical scoring criteria.
