# Supplementary material for: Passive Transfer of Animal-Derived Polyclonal Hyperimmune Antibodies Provides Protection of Mice from Lethal Lassa Virus Infection
Source: Viruses. 2023 Jun 26;15(7):1436. doi: 10.3390/v15071436 (PMC10384048; doi:10.3390/v15071436)
Supplement: Supplementary file 1 [file viruses-15-01436-s001.zip › Figure S1.pdf]

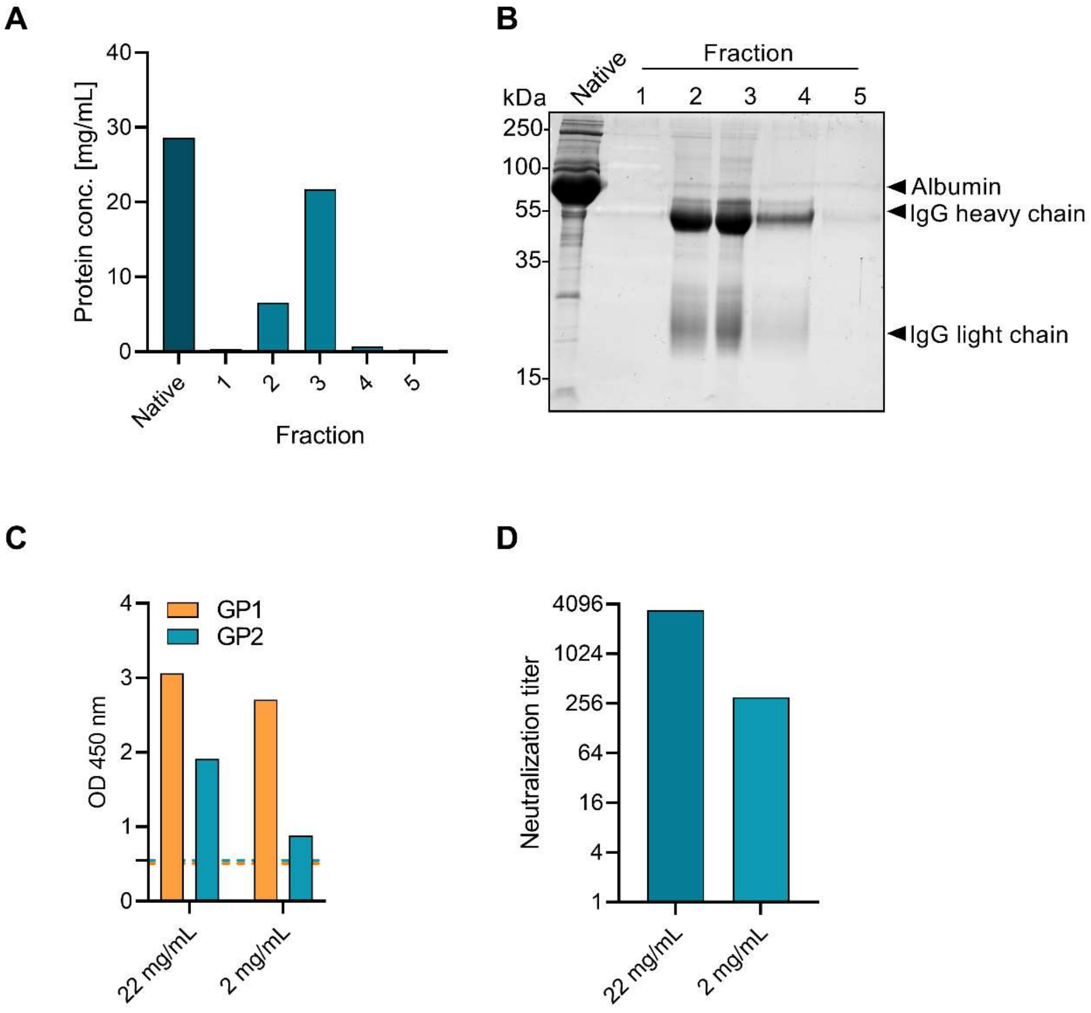

Figure S1. Purification and functional analysis of anti-LASV GP antibodies. **A** Total IgG was purified from rabbit hyperimmune sera using protein A affinity chromatography. The antibody concentration was determined using Pierce BCA Protein Assay Kit. **B** Purity of the IgG preparation was assessed by SDS-PAGE and SYPRO Ruby protein gel staining using 3 µg of total protein from each fraction. **C** IgG antibody binding responses against LASV GP were measured by GP subunit ELISA using GP1 and GP2 of LASV strain GA391 recombinantly expressed in HEK293 cells. **D** Neutralization titers of affinity-purified IgG were determined by replication-competent vesicular stomatitis virus (VSV) expressing LASV GP (VSVΔG/LASVGP).
