# Supplementary material for: Passive Transfer of Animal-Derived Polyclonal Hyperimmune Antibodies Provides Protection of Mice from Lethal Lassa Virus Infection
Source: Viruses. 2023 Jun 26;15(7):1436. doi: 10.3390/v15071436 (PMC10384048; doi:10.3390/v15071436)
Supplement: Supplementary file 1 [file viruses-15-01436-s001.zip › Table S1.pdf]

Supplementary Table S1: Overview of score points for daily monitoring of mice

Category	Description	Score	Humane end point
Appearance	Smooth fur	0	–
	Patches of hair piloerected	1	–
	Majority of fur piloerected	2	–
Level of consciousness	Mouse shows normal activity	0	–
	Mouse shows reduced activity	1	–
	Mouse only moves when provoked	2	–
	Mouse remains stationary when provoked	4	Yes
Eyes	Open, clear	0	–
	Milky secretion at one or both eyes	2	–
	Both eyes closed and milky secretion	3	–
Respiration	Normal	0	–
	Laboured	1	–
	Gasping	3	Yes
Weight loss	Weight loss <10% of starting weight	0	–
	Weight loss 10% – 19.9% of starting weight	1	–
	Weight loss greater 20% of starting weight	2	Yes
Neurological Abnormalities	Seizures, paralysis, disorientation, self–mutilation	3	Yes
Body temperature	Normal: 36.1 – 37.9 °C	0	–
	38.0 – 39.9 °C	1	–
	33.0 – 36.0 °C	1	–
	30.1– 32.9 °C	2	–
	≥40.0 °C	2	–
	≤30.0 °C	3	Yes
